# Rapid urease test (RUT) for evaluation of urease activity in oral bacteria in vitro and in supragingival dental plaque ex vivo

**DOI:** 10.1186/s12903-018-0541-3

**Published:** 2018-05-18

**Authors:** Gunnar Dahlén, Haidar Hassan, Susanne Blomqvist, Anette Carlén

**Affiliations:** Department of Oral Microbiology and Immunology, Institute of Odontology, Sahlgrenska Academy, University of Gothenburg, Box 450, SE 40530 Gothenburg, Sweden

**Keywords:** Rapid urease test, Supragingival dental plaque, Urease activity, Oral microbiota

## Abstract

**Background:**

Urease is an enzyme produced by plaque bacteria hydrolysing urea from saliva and gingival exudate into ammonia in order to regulate the pH in the dental biofilm. The aim of this study was to assess the urease activity among oral bacterial species by using the rapid urease test (RUT) in a micro-plate format and to examine whether this test could be used for measuring the urease activity in site-specific supragingival dental plaque samples ex vivo.

**Methods:**

The RUT test is based on 2% urea in peptone broth solution and with phenol red at pH 6.0. Oral bacterial species were tested for their urease activity using 100 μl of RUT test solution in the well of a micro-plate to which a 1 μl amount of cells collected after growth on blood agar plates or in broth, were added. The color change was determined after 15, 30 min, and 1 and 2 h. The reaction was graded in a 4-graded scale (none, weak, medium, strong). Ex vivo evaluation of dental plaque urease activity was tested in supragingival 1 μl plaque samples collected from 4 interproximal sites of front teeth and molars in 18 adult volunteers. The color reaction was read after 1 h in room temperature and scored as in the in vitro test.

**Results:**

The strongest activity was registered for *Staphylococcus epidermidis, Helicobacter pylori, Campylobacter ureolyticus* and some strains of *Haemophilus parainfluenzae*, while known ureolytic species such as *Streptococcus salivarius* and *Actinomyces naeslundii* showed a weaker, variable and strain-dependent activity. Temperature had minor influence on the RUT reaction. The interproximal supragingival dental plaque between the lower central incisors (site 31/41) showed significantly higher scores compared to between the upper central incisors (site 11/21), between the upper left first molar and second premolar (site 26/25) and between the lower right second premolar and molar (site 45/46).

**Conclusion:**

The rapid urease test (RUT) in a micro-plate format can be used as a simple and rapid method to test urease activity in bacterial strains in vitro and as a chair-side method for testing urease activity in site-specific supragingival plaque samples ex vivo.

## Background

Urease is an enzyme that hydrolyses urea (carbamide) into ammonia and carbon dioxide and is produced by several bacterial species. Detection of urease activity has become an important tool for the diagnosis of *Helicobacter pylori* infections in association with chronic gastritis, which increases the risk of developing peptic ulcer [1, 2]. Several oral bacterial species have been shown to produce urease e.g. *Streptococcus salivarius, Actinomyces naeslundii, Haemophilus parainfluenzae* [3, 4] although the understanding on the variation in the activity between species and strains is limited. Furthermore, the extent of urease production in vivo by the dental plaque bacteria is still unclear.

Urea is delivered in the gingival crevicular fluid (GCF) and in all salivary gland secretions at concentrations ranging from 3 to 10 mM in healthy individuals [5, 6]. Several studies have shown that urea in such concentrations can increase the baseline pH of the dental biofilm (plaque) and may significantly counteract the effects of glycolytic acidification in the plaque [7, 8]. Ammonia could also be produced by the hydrolysis of a number of amino acids. The net pH change in plaque is not only a result of ureolysis but other factors are also involved [8]. Urease activity in the plaque in situ has been measured indirectly by quantifying the amount of ammonia formed using Nessler’s reagent [9–11]. Nessler’s reagent is slow and estimates the ammonia concentration as well as the urease activity indirectly, however it is not suitable as a chair side method.

The rapid urease test (RUT) is a simple method used for the diagnosis of *H. pylori* infections [12] and which also has been used for measuring the urease activity in dental plaque samples ex vivo. In such studies focusing on the presence of *H. pylori* in the dental plaque, it was argued that *H. pylori* were the only dental plaque bacteria able to rapidly produce detectable amounts of urease [13]. The knowledge of the extent and rate of urease production in oral bacteria is limited. The first aim of this study was to use RUT to screen and grade the urease activity in oral bacterial species/strains in vitro. For that purpose, the RUT method was modified into a micro-plate format for the semi-quantification of bacterial urease activity by visual scoring. The second aim was to test if this RUT method could be used as a simple and rapid chair-side test to screen for site differences of urease activity in ex vivo plaque.

## Methods

### Bacteria

As seen in Table 1, the bacterial strains used for in vitro evaluation of RUT included both laboratory reference strains (Culture Collection, University of Gothenburg, CCUG, Sweden; American Type Culture Collection, ATCC) and own clinical isolates (Oral Microbiology, Gothenburg, Sweden, OMGS). *Campylobacter ureolyticus*, *H. pylori, Staphylococcus epidermidis* strains were used as positive controls and *Enterococcus faecalis, Escherichia coli*, *Pseudomonas aeruginosa* and *Staphylococcus aureus* strains as negative controls according to their known present or absent ureolytic activity [14]. In addition, oral bacterial species e.g. *A. naeslundii, H. parainfluenzae* and *S. salivarius* with documented ureolytic capacity were included [3, 4]. Furthermore, we also included a number of strains commonly associated with supragingival and subgingival dental plaque (see Table 1). Fresh clinical isolates of *A. naeslundii, H. parainfluenzae* and *S. salivarius* from saliva samples in the oral microbiological diagnostic laboratory of the department, identified using common laboratory methods [14], were used for evaluation of bacterial strain variability (Table 2).

**Table 1:** Bacterial species tested for urease activity with the RUT test, read after 1 h incubation at 36 °C

Bacterial species	Origin or strain designation^a^	Urease activity^b^
*Actinomyces naeslundii*	OMGS 2466	+
*Actinomyces naeslundii*	OMGS 1923	0
*Actinomyces oris*	OMGS 2683	+
*Campylobacter gracilis*	CCUG 27720	+
*Campylobacter rectus*	OMGS 1236	+
*Campylobacter ureolyticus*	CCUG 7319	+++
*Enterococcus faecalis*	ATCC19433	0
*Escherichia coli*	OMGS 3935	0
*Fusobacterium nucleatum*	OMGS 2685	0
*Haemophilus parainfluenzae*	CCUG 12836 T	0
*Haemophilus. parainfluenzae*	OMGS 199/11	++
*Haemophilus parainfluenzae*	OMGS 202/11	+++
*Haemophilus parainfluenzae*	OMGS 203/11	+++
*Helicobacter pylori*	ATCC 43504	+++
*Lactobacillus casei/paracasei*	OMGS 3184	0
*Lactobacillus. salivarius*	CCUG 55845	0
*Lactobacillus fermentum*	OMGS 3182	+
*Porphyromonas gingivalis*	OMGS 2860	0
*Prevotella intermedia*	OMGS 2514	0
*Pseudomonas aeruginosa*	OMGS 3943	0
*Rothia dentocariosa*	OMGS 1956	0
*Streptococcus mitis*	CCUG 31611	+
*Streptococcus mutans*	OMGS 2482	0
*Streptococcus salivarius I*	OMGS 3944	0
*Streptococcus salivarius II*	OMGS 3945	+
*Streptococcus sanguinis*	OMGS 2478	0
*Staphylococcus aureus*	OMGS 3947	+
*Staphylococcus epidermidis*	OMGS 3949	+++
*Tannerella forsythia*	ATCC43037	0

^a^ATCC and CCUG means reference strains, OMGS means clinical isolates from the department collection

^b^The visual color change was graded as no reaction (0) for no color change, + denoted a weak reaction with a shift to pink color, ++ denoted a moderate reaction with a shift towards red and +++ a strong reaction with a clear purple color

**Table 2:** Reactions from the clinical oral isolates in the RUT method read after 1 h. Cells were obtained from agar plates by scraping (1 μl)

Bacteria	No reaction No of strains (%)	Weak No of strains (%)	Moderate No of strains (%)	Strong No of strains (%)
*Streptococcus salivarius* (*N* = 8)	4 (50%)	2 (25%)	2 (25%)	0 (0%)
*Actinomyces naeslundii* (*N* = 8)	7 (87.5%)	1 (12.5%)	0 (0%)	0 (0%)
*Haemophilus. parainfluenzae* (*N* = 5)	1 (20%)	0 (0%)	0 (0%)	4 (80%)

### Urease test

The NCTC micro method (National Collection of Type Cultures, NCTC, Public Health England, UK) of RUT used for detection of *H. pylori* in stomach samples [15, 16] was modified into a micro-plate format and used for the estimation of urease positive bacteria. Urease-broth (Bakt lab, Sahlgrenska hospital, Gothenburg, Sweden) was used in volumes of 100 μl in 96-hole micro-titer plates (Nunc, Copenhagen, Denmark). The nutrient broth contained 2% urea, pH 6.8, and phenol red as an indicator. The broth has an orange color, which turns yellow at a lower pH and pink to red then purple at alkaline pH.

### In vitro evaluation of urease activity in bacterial strains

The bacterial strains shown in Table 1 (including the urease positive and negative control strains) were grown on Brucella-blood agar plates aerobically for 2 days or, in the case of anaerobic species, anaerobically for 5–7 days at 36^o^ C. Colonies from the plates were harvested by scraping a loopful (1 μl Inoculation loop, Sarstedt, Nümbrecht, Germany) of colony cells, which were suspended into the urea-broth and incubated at 36 °C. The color change was read after 15 min, and 30 min, and 1 and 2 h. The visual color change was graded as no reaction (0) for no color change (or noted as ‘changed to yellow as a result of acid production’), + denoted a weak reaction with a shift to pink color, ++ denoted a moderate reaction with a shift towards red and +++ a strong reaction with a clear purple color.

*S. epidermidis* (OMGS 3949), *C. ureolyticus* (CCUG 7319), *S. salivarius* (OMGS 3945) and *A. naeslundii* (OMGS 2466) were used as positive controls, and *E. coli* (OMGS 3935) as a negative control, in experimental series performed in order to further evaluate the method’s dependency on time and temperature. To test the time dependency, the plates were left on the bench and were read every hour for another 4 h and finally after 24 h. The temperature dependency was tested at similar time points after incubation of the plates at the temperature’s 20, 25 and 36 °C. All experiments were performed in triplicate.

The same bacterial strains were used to test RUT for the dependence of glucose and lowered pH during the bacterial growth before inoculation in the urea test broth. The strains were incubated in a peptone broth containing 0.1, 0.5 or 1.0% glucose for 1–2 days. The final pH was measured and the bacterial cells were harvested by centrifugation and a loopful (1 μl) amount was then used to test the urease activity with the RUT method. The reaction was followed and read after 1 h.

The strain variability in urease activity was tested by using fresh clinical isolates of *S. salivarius* (8 strains), *A. naeslundii* (8 strains) and *H. parainfluenzae* (5 strains) and using both cells obtained from agar plates (1 μl) and from a pellet (1 μl) after broth culture.

### Ex vivo evaluation of urease activity in dental plaque

Eighteen volunteers (11 females and 7 males, mean age ± SD of 37.3 ± 15.4, range 25–69) among students and laboratory personnel at the Institute of Odontology, University of Gothenburg, participated in the test. They had a DMFT±SD (Decayed Missed Filled Teeth ±Standard deviation) of 9.7 ± 5.54. No further specific inclusion criteria were considered necessary for evaluating the RUT method on dental plaque ex vivo. The subjects were instructed not to brush their teeth 2 days prior to the test and not to eat or drink anything except water 2 h prior to the test. They all participated voluntarily and gave an informed consent.

Individual supragingival plaque samples were collected by scraping with a curette from interproximal sites between the lower incisors (site 31/41), between the upper incisors (site 11/21), between the upper left first molar and second premolar (site 26/25), and between lower right second premolar and molar (site 45/46) on 18 adult individuals. A loopful (1 μl) amount of plaque from each respective site was added to 100 μl of urea broth in a 96-hole plate and left in room temperature. The color reaction was read after 1 h and was graded similar to the bacterial in vitro tests.

### Statistical analysis

Non-parametric Mann-Whitney test were used to analyse the difference of urease activity of the interproximal supragingival plaque between the sites. The statistical analysis were performed using Microsoft® Excel® for Mac (Version 14.4.8, 2011) and KaleidaGraph® (version 4.1.2, Synergy Software. 2011). It was considered statistically significant if the *p*-value was < 0.05.

## Results

### Urease evaluation in vitro

Distinct reactions were obtained after 1 h at 36 °C using bacteria grown on agar plates (Table 1). Strong, purple and rapid reactions (+++) were seen for *C. ureolyticus*, *H. parainfluenzae* (two strains), *H. pylori* and *S. epidermidis,* which had already turned purple after 15 min*.* One strain of *H. parainfluenza* showed a slower, moderate reaction (++) whereas another showed no reaction after 1 h. Weak and pink color reactions (+) only were registered for some strains of *Actinomyces* spp., *Campylobacter* spp*.*, *Lactobacillus* spp*.,* alpha-streptococci and *S. aureus*. No reaction (0) was obtained from the anaerobic periodontal disease associated species; e.g. *Porphyromonas gingivalis, Tannerella forsythia, Fusobacterium nucleatum* and *Prevotella. intermedia* or opportunistic strains tested; e.g. *P. aeruginosa*, *E. faecalis* and *E. coli*.

**Fig. 1:**
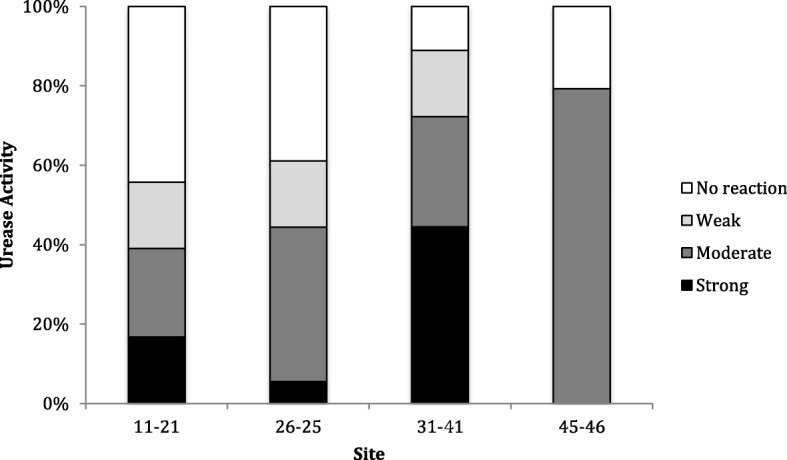
Urease activity (% of samples) in plaque samples from between upper (interproximal site 11/12) and lower (interproximal site 31/41) central incisor and from the mesial aspect of upper left (interproximal site 26/25) and lower right (interproximal site 45/46) first molar of 18 adult volunteers

The time dependency tests showed that both slow and rapid urease-producing bacteria could clearly be distinguished after 1 h incubation in the RUT-medium. Strong positive strains (*H. pylori, C. ureolyticus*, *S.epidermidis* and *H. parainfluenzae*) showed a color change to purple within 15 min. The color change was otherwise gradual and slow for urease positive oral strains with minor, insignificant changes from 1 h to 2 h. Bacterial strains that did not change colors within 2 h were registered as negative. From these findings it was decided to read the reactions after a fixed time (1 h) for the strains (Table 1) and plaque samples (Fig. 1). Moreover, the effect of temperature (20, 25, or 36 °C) was only marginal on the color reaction (data not shown) and, thus, the final color reaction was read after 1 h at room temperature in all further in vitro and ex vivo experiments.

The urease activity in cells cultured in broth with a glucose concentration < 1% was generally weaker than in cells after plate culture. The influence of pH during growth was found to be negligible in the interval between 6.0 and 7.5 but a yellow color developed for some streptococcal strains with a pH < 5.0 in the broth before harvesting.

Tests using several strains of the same species showed strong urease activity after 1 h (+++) in 4 strains (80%) of *H. parainfluenzae* and a moderate (++) and a weak reaction (+) in 2 strains (25%) respectively of *S. salivarius*. No reaction (0) was seen for 7 of the 8 strains of *A. naeslundii* tested, while one strain showed a weak positive reaction. The result was the same regardless if the cells were grown on agar or in broth (Table 2) and illustrates the phenotypic variability between the strains of the same species.

#### Ex vivo urease plaque test

Interproximal supragingival plaque samples collected from the interproximal site of the lower central incisors (31/41) showed significantly higher urease activity than samples from the interproximal sites of upper central incisors (11/12) (*p* < 0.05), upper left first molar and premolar (26–25) (*p* < 0.05) and lower right first molar and premolar (45–46) (*p* < 0.001). The differences between the three latter sites were not significant. The frequency of the RUT scores for each of the 4 sites is shown in Fig. 1.

## Discussion

Urea agar or broth test has been used routinely in bacteriological laboratories to test bacterial strains for urease activity. The broth test is described in most manuals in clinical microbiology [14]. It is a simple, reliable, and rapid test that is used in vivo*/*ex vivo for diagnostic purpose to detect urease positive *H. pylori* in peptic ulcers [15]. For the detection of *H. pylori* infections, both in-house and commercial variants of RUT have been developed [15–17]. The present study found that a NCTC modified micro-titer plate method of the rapid urease test (RUT) could be used to screen and semi-quantify the urease activity among bacterial strains in vitro and as a chair-side method for assessing the site specific ureolytic activity in dental plaque samples ex vivo.

A strong reaction was noticed already after 15 min for bacteria with the most expressed urease activity such as *H. pylori, C. ureolyticus* and *S. epidermidis,* which are not normally considered as resident in the dental plaque. Some oral streptococci e.g. *S. salivarius* and *S. mitis* showed weak urease activity. Others like *A. naeslundii*, more commonly found in the dental plaque also showed a weak activity. Both streptococci and *Actinomyces* strains needed 1–2 h to show some reaction. Longer incubation time may affect the outcome of the test due to bacterial growth and acid production from sugars or ammonia production by amino acid degradation (arginolysis). One hr. was therefore chosen as a reliable incubation time that could be used also for chair-side tests. Furthermore, tests performed at 20, 25 and 36 °C showed that temperature had a limited effect on the urease reaction and there was no difference between the results obtained from plate and broth cultured bacterial cells. Also, except for strong acidogenic species giving slightly yellow color in the in vitro test if the cell material used for inoculation had a low pH (≤ 5), similar results were obtained from cells grown with or without glucose before the RUT test. These findings suggest that the production of urease by bacteria is a stable and conserved characteristic for many bacterial species, which is generally not affected by environmental factors such as temperature and the presence of glucose or the broth. They further suggest that for ex vivo estimation of the plaque urease activity, RUT should be used on patients who have abstained from sugar-containing food or drinks for at least 2 h. Also, the amount of bacterial cells and plaque material for ex vivo test using RUT, may affect the outcome. There is, however, no ideal and standardized way to collect dental plaque. We evaluated the use of a loopful (1 μl) and found the amount to give minor, non-significant variations only when repeated in triplicates.

Many oral bacteria are described in the literature as being ureolytic. Much focus has been paid on *S. salivarius* and *A. naeslundii* [8], which have been claimed to play an important role in the dental plaque ecology by reducing the plaque acidity and thereby having anti-cariogenic effects [7, 18–20]. Other streptococci have not been shown to have this capacity. Most of the referred studies are performed in models in vitro and the phenotypic expression of the involved genes encoding for the enzyme activity may vary due to the environment [21–23]. This may be one reason why the ureolytic capacity was not a common feature for the phenotype of the strains of *S. salivarius* and *A. naeslundii* tested in the present study. The low activity of the *A. naeslundii* strains may be explained by several taxonomic revisions of this species (previously named Genospecies I and II) [24, 25]. Genospecies II (previously called *A. viscosus*) is now called *A. oris* and the strain tested here showed a positive reaction, while those classified as *A. naeslundii* had a weaker capacity.

For *H. parainfluenzae*, which are not implicated in caries disease but more associated with gingivitis [26, 27], 4 out of 5 strains showed a strong and rapid urease activity (Table 2). No urease activity seen for one strain may be due to different biotypes. In a recent publication, strains of the genus *Haemophilus* and especially *H. parainfluenzae* were significantly more prevalent in the dental plaque of children with a high urease activity and it was concluded that they were major contributors of the urease enzyme for the alkali production in the plaque [28]. Thus, previous and present findings suggest that *H. parainfluenzae* may be of greater importance to the alkalization of the dental plaque through ureolysis than streptococci and *Actinomyces* spp.

It is worth notice that *F. nucleatum, P. gingivalis, P. intermedia* and *T. forsythia,* all associated to periodontal disease were negative for ureolytic activity. This is of no surprise due to the fact that the periodontal pocket normally is slightly alkaline [29], and there is less need for the acid intolerant bacteria to neutralize acids compared to the acidogenic supragingival environment.

It is important to note that also some other bacterial species such as *Campylobacter* spp. (*C. ureolyticus* in particular) and *H. pylori* showed a rapid ureolytic activity, giving a positive purple reaction within 15 min. They are normally considered to belong to the gastro-intestinal and not to the oral microbiota. However, *C. ureolyticus* as well as *H. pylori* are intermittently found in the dental plaque from the subgingival area and in periodontal diseases when DNA probes or PCR methodology are used for the analyses [30, 31]. The strong and rapid reaction of these two bacteria indicate a putative significant contribution to the net urease activity in the plaque, even when present in lower numbers compared to other but predominant plaque bacteria, with a considerably lower urease activity. According to a recently published review the RUT method has also been used for the detection of *H. pylori* in dental plaque [13]. In view of our finding of many urease positive species that can be present in the dental plaque, this must be considered doubtful.

One advantage with the RUT method applied for plaque samples is the possibility to use it site-specifically. The screening of four different sites in a group of adult individuals showed a strong activity in the mandibular anterior region of > 70% of the individuals. This activity was significantly higher than in plaque samples from the other sites of the dentition tested. It is well known that the teeth in the mandibular anterior region are characterized by a low caries experience and high prevalence of calculus, related to low and high pH, respectively. Ureolysis may therefore have a biological impact on both periodontitis and caries. This study did, however, not consider the clinical status of the included individuals. It did, however, show that the method could be used as a chair side method for site-specific urease activity measurements, which could be of interest for the evaluation the individual susceptibility to dental diseases.

## Conclusions

This study evaluated the usefulness of a simple test (Rapid urease test, RUT) for the assessment and semi-quantification of the ureolytic activity in various bacterial species in vitro as well as in dental plaque samples ex vivo. Bacteria with strong, moderate, weak or no activity were distinguished. Strains of *Haemphilus parainfluenzae*, but not of other common plaque bacteria tested, showed strong and rapid urease activity. Dental plaque from mandibular anterior teeth frequently showed a significantly higher urease activity ex vivo than plaque from other sites. It can be concluded that the RUT method can be used as a simple and rapid method in order to assess urease activity in bacteria in vitro and in plaque samples ex vivo.

## Abbreviations

ATCC: American Type Culture Collection
CCUG: Culture Collection University of Gothenburg
DMFT: Decayed Missed Filled Teeth
GCF: Gingival crevicular fluid
NCTC: National Collection of Type Culture
OMGS: Oral Microbiology Gothenburg Sweden
RUT: Rapid urease test

## References

[1] Wildner-Christensen M, Lassen AT, Lindebjerg J, Schaffalitsky De Muckadell OB (2002). Diagnosis of *Helicobacter pylori* in bleeding peptic ulcer patients, evaluation of urea-based tests. Digestion.

[2] Tang JH, Liu NJ, Cheng T (2009). Et al endoscopic diagnosis of Helicobacter infection by rapid urease test in bleeding peptic ulcers: a prospective case-control study. J Clin Gastroenterol.

[3] Salako NO, Kleinberg I (1989). Incidence of selected ureolytic bacteria in human dental plaque from sites with differing salivary access. Arch Oral Biol.

[4] Chen YY, Weaver CA, Burne RA (2000). Dual functions of *Streptococcus salivarius* urease. J Bacteriol.

[5] Golub LM, Borden SM, Kleinberg I (1971). Urea content of gingival crevicular fluid and its relation to periodontal diseases in humans. J Periodontal Res.

[6] Al-Nowaiser A, Roberts GJ, Trompeter RS, Wilson M, Lucas VS (2003). Oral health in children with chronic renal failure. Pediatr Nephrol.

[7] Kleinberg I (1967). Effect of urea concentration on human plaque in situ. Arch Oral Biol.

[8] Bowen WH (2013). The Stephan curve revisited. Odontology.

[9] Shu M, Morou-Bermudez E, Suarez-Perez E, Rivera-Miranda C, Browngardt CM, Chen Y-YM, Magnusson I, Burne RA (2007). The relationship between dental caries status and dental plaque urease activity. Oral Microbiol Immunol.

[10] Nascimento MM, Gordan VV, Garvan CW, Browngardt CM, Burne RA (2009). Correlation of oral bacterial arginine and urea catabolism with caries experience. Oral Microbiol Immunol.

[11] Toro E, Nascimento MM, Suarez-Perez E, Burne RA, Elias-Boneta A, Morou-Bermudez E (2010). The effect of sucrose on plaque and saliva urease levels in vivo. Arch Oral Biol.

[12] Goh KL, Parasakthi N, Peh SC, Puthucheary SD, Wong NW (1994). The rapid urease test in the diagnosis of *Helicobacter pylori* infection. Singap Med J.

[13] Anand PS, Kamath KP, Anil S (2014). Role of dental plaque, saliva and periodontal disease in *Helicobacter pylori* infection. World J Gastroenterol.

[14] Barrow GI, Feltham RKA (2004). Cowan and Steel’s manual for the identification of medical bacteria.

[15] Said RM, Cheah P-L, Chin S-C, Goh K-L (2004). Evaluation of a new biopsy urease test: Prontodry, for the diagnosis of *Helicobacter pylori* infection. Eur J Gastroenterol Hepatol.

[16] Boromeo M, Lambert JR, Pinkard KJ (1987). Evaluation of the “CLO-test” to detect *Campylobacter pyloridis* infection. J Clin Patol.

[17] Levin DA, Watermayer G, Mohamed N, Epstein DP, Hiatshwayo SJ, Metz DC (2007). Evaluation of a locally produced rapid urease test for the diagnosis of *Helicobacter pylori* infection. S Afr Med J.

[18] Hassan H, Lingström P, Carlen A (2015). Plaque pH in caries-free and caries-active young individuals before and after frequent rinses with sucrose and urea solution. Caries Res.

[19] Biswas SD (1982). Effect of urea on pH, ammonia, amino acids and lactic acid in the human salivary sediment system incubated with varying levels of glucose. Arch Oral Biol.

[20] Sissons CH, Hancock EM (1993). Urease activity in *Streptococcus salivarius* at low pH. Arch Oral Biol.

[21] Yaling L, Tao H, Jingyi Z, Xuedong Z (2006). Characterization of the *Actinomyces naeslundii* ureolysis and its role in bacterial aciduricity and capacity to modulate pH homeostasis. Microbiol Res.

[22] Morou-Bermudez E, Burne RA (2000). Analysis of urease expression in *Actinomyces naeslundii* WVU45. Infect Immun.

[23] Liu Y, Hu T, Jiang J, Zhou X (2008). Regulation of urease gene of *Actinomyces naeslundii* in biofilms in response to environmental factors. FEMS Microbiol Lett.

[24] Johnson JL, Moore LVH, Kaneko B, Moore WEC (1990). *Actinomyces georgiae* sp. Nov. *Actinomyces gerencseriae* sp. Nov., designation of two genospecies of *Actinomyces naeslundii*, and inclusion of *A. naeslundii* serotypes II and III and *Actinomyces viscosus* serotype II in *A. naeslundii* genospecies 2. Int J Syst Bacteriol.

[25] Henssage U, Do T, Radford DR, Gilbert SC, Clark D, Beighton D (2009). Emended description of *Actinomyces naeslundii* and descriptions of *Actinomyces oris* sp. Nov. and *Actinomyces johnsonii* sp. Nov., previously identified as *Actinomyces naeslundii* genospecies 1,2 and WVA 963. Int J Syst Evolution Microbiol.

[26] Liljemark WF, Bloomquist CG, Uhl LA, Schaffer EM, Wolff LF, Pihlstrom BL, Bandt CL (1984). Distribution of oral Haemophilus species in dental plaque from a large adult population. Infect Immun.

[27] Socransky SS, Haffajee AD (2000). Periodontal microbial ecology. Periodontol.

[28] Morou-Bermudez E, Rodriguez S, Bello AS, Dominguez-Bello MG (2015). Urease and dental plaque microbial profiles in children. PLoSONE.

[29] Marsh PD, Martin MV (2009). Oral Microbiology.

[30] Appelgren L, Dahlen A, Eriksson C, Suksuart N, Dahlen G (2014). Dental plaque pH and ureolytic activity in children and adults of a caries population. Acta Odont Scand.

[31] Bouziane A, Ahid S, Abougal R, Ennibi O (2012). Effect of periodontal therapy on prevention of gastric *Helicobacter pylori* recurrence: a systematic review and meta-analysis. J Clin Periodontol.

